# Open cavotomy for retrieval of inferior vena cava cement embolism after cement-augmented spinal fusion

**DOI:** 10.1016/j.jvscit.2026.102184

**Published:** 2026-02-12

**Authors:** Ivan Sokolik, Laura Zimmermann, Alexis Paoli, Sébastien Déglise

**Affiliations:** Department of Vascular Surgery, Centre Hospitalier Universitaire Vaudois (CHUV), Lausanne, Switzerland

**Keywords:** Cement-augmented spinal surgery, Cement embolism, Inferior vena cava, Open cavotomy, Vertebroplasty complications

## Abstract

Cement leakage during cement-augmented spinal procedures is common, but venous cement embolism remains an uncommon and potentially serious complication. We report the case of an 82-year-old woman who developed a large cement embolus partially occluding the inferior vena cava after cement-augmented spinal surgery. Postoperative imaging demonstrated a mobile intraluminal fragment at the level of the left renal vein, with additional smaller asymptomatic emboli. Given the size and mobility of the embolus, open surgical retrieval through cavotomy was performed with successful en bloc removal. This case highlights that open cavotomy is a safe option for selected patients with high-risk venous cement emboli.

Although cement leakage during percutaneous vertebroplasty has been reported in up to three-quarters of cases, venous cement embolism remains an uncommon but clinically significant complication.^1^^,^^2^ Venous intravasation occurs in up to 24% of vertebroplasty procedures, and pulmonary cement embolism is detected in 3% to 23% of cases when actively screened using computed tomography (CT).^2^^,^^3^ Although most emboli remain asymptomatic and can be managed conservatively, larger or centrally located fragments may lead to severe cardiopulmonary complications requiring vascular surgical intervention.^3^^,^^4^ Prompt imaging and multidisciplinary assessment are therefore essential to guide management.

Several case reports have described unusual presentations and management strategies. Dash and Brinster reported an intracardiac polymethylmethacrylate (PMMA) embolus requiring median sternotomy and cardiopulmonary bypass.^4^ Agko et al described prophylactic inferior vena cava (IVC) filter placement during kyphoplasty, which allowed subsequent percutaneous retrieval of a cement fragment.^5^ Duran and colleagues reported successful open cavotomy for removal of a large cement cast from the intrahepatic IVC.^6^ These reports highlight the wide spectrum of cement embolism and the need for tailored vascular approaches.

We report a rare but potentially life-threatening case of a large IVC cement embolism in an elderly woman following cement-augmented spinal surgery.

Written informed consent for publication of this case and accompanying images was obtained from the patient.

## Case report

### Patient presentation and preoperative assessment

An 82-year-old Caucasian woman, known for hypertension, supraventricular tachycardia, chronic nutritional deficiency, and severe postmenopausal osteoporosis, with a history of L3 to L5 posterior decompression performed 15 years earlier, presented with acute worsening of chronic mid-lumbar pain following the lifting of a light gardening tool. Her previous surgery had been complicated by a residual mild left L5 paresis. The pain was sharp, exacerbated by flexion and extension, and partially relieved by rest and paracetamol. She denied new motor deficits, sensory changes, or bladder and bowel dysfunction.

Lumbar magnetic resonance imaging revealed an acute osteoporotic fracture of the L3 vertebral body (OF4 classification) with approximately 25% anterior height loss, as well as recurrent severe central canal stenosis at L4 to L5 caused by a large posterior disc herniation compressing both L5 nerve roots. Moreover, an unstable L4 to L5 spondylolisthesis associated with advanced facet arthropathy was also diagnosed. A combined surgical approach was planned: percutaneous balloon kyphoplasty of L3, posterior decompression at L4 to L5 with dural repair, and cement-augmented pedicle screw fixation from L4 to L5.

### Spinal surgery and postoperative evolution

Under general anesthesia with neuromonitoring, balloon kyphoplasty of L3 was performed via bilateral transpedicular access under biplanar fluoroscopy, with the patient in the prone position. PMMA cement was injected only once a toothpaste-like viscosity was achieved, with a total volume of 4 mL per side. Subsequently, posterior decompression and instrumentation were performed through a midline approach. Pedicle screws were inserted at the L4 and L5 levels, and a small incidental dural tear was repaired primarily and reinforced with a collagen patch. Cement augmentation (1 mL per screw) was carried out via fenestrated pedicle screws under fluoroscopic control. Toward the end of cement injection, fluoroscopic image quality deteriorated due to bowel gas and suboptimal positioning, revealing a linear hyperdense structure at the L1 to L2 level, suspicious for venous cement leakage. Cement injection was immediately stopped. The patient remained hemodynamically stable throughout the procedure.

**Fig 1:**
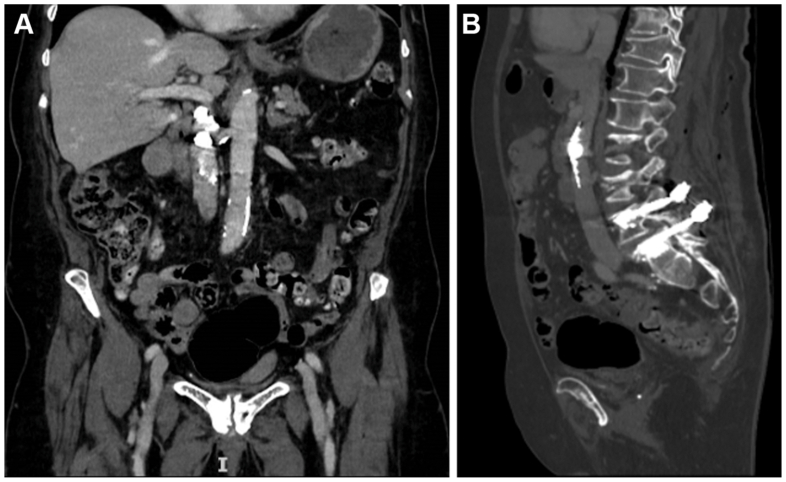
**(A)** Contrast-enhanced coronal computed tomography (CT) image showing a large linear cement cast partially occluding the infrahepatic inferior vena cava (IVC) at the level of the left renal vein. **(B)** Noncontrast sagittal CT reconstruction demonstrating the elongated cement cast extending cranially from the lumbar vertebral level.

A noncontrast CT obtained on postoperative day 1 demonstrated a large linear cement cast lodged within the infrahepatic IVC at the level of the left renal vein ostium, which was partially occluded (Fig 1). Two smaller cement fragments were also identified within the left hepatic vein and a superior lobar pulmonary artery. The patient remained asymptomatic, with stable vital signs and no cardiopulmonary complaints. Duplex ultrasound confirmed a partially mobile echogenic intraluminal mass within a patent IVC, associated with mildly increased flow velocity but no obstruction or thrombosis. Transthoracic echocardiography showed normal right ventricular function and no intracardiac emboli.

### Vascular surgical management

**Fig 2:**
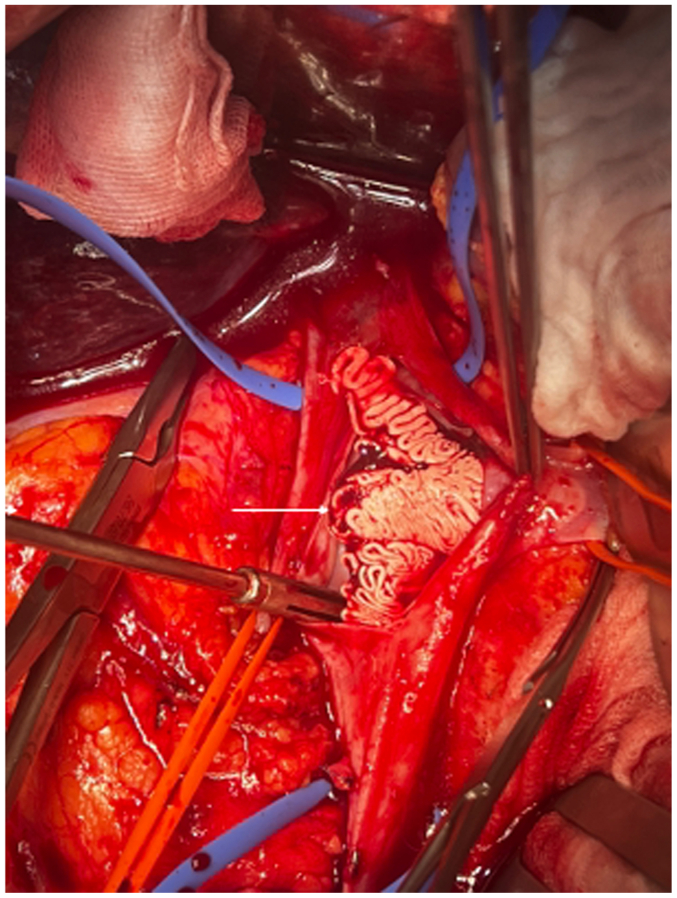
Intraoperative view during open cavotomy demonstrating en bloc removal of a nonadherent cement cast from the inferior vena cava (IVC) (*arrow*).

Given the size and partial mobility of the IVC cement cast, as well as the risk of fragmentation and fatal pulmonary embolism, conservative treatment was considered to carry a high risk of complications, and cast retrieval was therefore decided. As endovascular snaring of this large fragment was deemed unrealistic and unsafe, as were other endovascular attempts, open surgical removal was selected following multidisciplinary discussion. Three days after spinal surgery, the patient underwent midline laparotomy. After mobilization of the right colon and duodenum, the infrahepatic IVC and left renal vein confluence were carefully exposed. Following systemic heparinization, proximal and distal vascular control was achieved, and a longitudinal cavotomy was performed (Fig 2). The PMMA cement cast, which was not adherent to the venous wall, was easily removed en bloc. The IVC was then closed primarily using a running 5-0 Prolene suture. Clamping time was 17 minutes, and blood loss was minimal.

The patient recovered uneventfully and was discharged to a rehabilitation facility on postoperative day 13. Given the small and asymptomatic retained cement fragments in the hepatic and pulmonary vascular beds, conservative management with oral anticoagulation was initiated for 3 months. At 1-month follow-up, she remained asymptomatic, with stable imaging findings.

## Discussion

Considering the aging population, the rate of elderly people suffering from degenerative spinal disease and osteoporosis is increasing. Percutaneous vertebral cement augmentation is considered a reliable method and has transformed the management of painful osteoporotic compression fractures, yet cement leakage remains its most common complication.^2^^,^^7^ Venous intravasation occurs due to high-pressure injection into the valveless vertebral venous system, which provides a direct pathway to the azygos system and IVC.^8^ Low cement viscosity, high injection pressure, large cement volumes, multilevel procedures, and severe osteoporosis all increase the risk of embolization.^9^^,^^10^ Moreover, vertebral treatment at the thoracic level seems to be associated with a higher risk of pulmonary embolism. Accordingly, several technical measures have been described to minimize the risk of venous intravasation during cement injection, including careful confirmation of needle positioning, avoidance of venous backflow on aspiration prior to injection, use of adequately viscous cement, and slow incremental injection under continuous biplanar fluoroscopy.

Preventive measures include delaying injection until adequate cement viscosity is achieved, using small incremental injections under continuous biplanar fluoroscopy, and terminating injection immediately upon detection of leakage.^9^^,^^11^ Balloon kyphoplasty reduces intravertebral pressure compared with vertebroplasty, potentially lowering the risk of cement leakage. Adjunctive techniques such as venography or prophylactic IVC filter placement have been described but remain controversial.^5^^,^^12^ In most cases, IVC filters have been placed to prevent pulmonary embolism after the diagnosis of deep vein thrombosis and not to prevent cement embolization. Therefore, the risk-benefit ratio for prophylactic IVC filter placement remains unclear. In the present case, placement of a suprarenal IVC filter combined with anticoagulation was considered. However, given the location of the cement cast at the level of the renal veins with partial involvement of the left renal vein ostium, suprarenal filter placement was felt to carry an increased risk of filter-related complications and would not have addressed the risk of progressive renal vein obstruction. Therefore, primary removal of the cement cast was favored.

Routine postoperative CT screening is not recommended, and no guidelines exist for a specific work-up; however, when cement migration is suspected, prompt imaging is essential to confirm the diagnosis and evaluate the extent of the migration.^13^ Furthermore, CT remains the gold standard for localization, whereas duplex ultrasound and echocardiography provide additional functional assessment.

In the absence of clear guidelines, management strategies for cement extravasation vary and depend on fragment size, location, and symptomatology. Small, peripheral emboli are often managed conservatively. As small emboli may remain asymptomatic or become symptomatic later, the exact rate of these complications is underestimated. To avoid additional clot formation around the cement cast, anticoagulation is generally recommended.^3^^,^^14^ In cases of symptomatic embolism or when the fragments are considered at high-risk of further complications such as perforation or central occlusion, removal is justified. Endovascular retrieval may be feasible for small, mobile fragments, whereas rigid or large casts typically require open surgical removal.^2^^,^^4^^,^^6^ Although large-bore aspiration catheters can be considered for selected venous cement emboli, this approach was deemed unsuitable in the present case due to the large size, rigidity, and cast-like morphology of the cement fragment, which would have rendered aspiration unlikely even with large-bore devices. In addition, the partial mobility of the embolus, its proximity to the left renal vein ostium, and the associated risk of fragmentation and uncontrolled embolization during manipulation, further supported the decision for open cavotomy, which provided safe and definitive treatment.

## Conclusions

Venous cement embolism is an uncommon but potentially serious complication of cement-augmented spinal procedures. Management should be individualized according to embolus size, location, and mobility. This case illustrates that open cavotomy is a safe and effective option for selected patients with large or high-risk IVC cement emboli.

## Declaration of generative AI and AI-assisted technologies

The authors used generative AI to assist with English-language editing and clarity. The authors reviewed and edited the content and take full responsibility for the final manuscript.

## Funding

None.

## Disclosures

None.
